# Revealing the Role of Sidewall Orientation in Wet Chemical Etching of GaN-Based Ultraviolet Light-Emitting Diodes

**DOI:** 10.3390/nano9030365

**Published:** 2019-03-05

**Authors:** Hui Wan, Bin Tang, Ning Li, Shengjun Zhou, Chengqun Gui, Sheng Liu

**Affiliations:** 1 The Institute of Technological Sciences, Wuhan University, Wuhan 430072, China; wanhui_hb@whu.edu.cn (H.W.); cheng.gui.2000@gmail.com (C.G.); Shengliu@whu.edu.cn (S.L.); 2 Center for Photonics and Semiconductors, Hubei Key Laboratory of Waterjet Theory and New Technology, School of Power and Mechanical Engineering, Wuhan University, Wuhan 430072, China; bintang@whu.edu.cn (B.T.); liningmick@whu.edu.cn (N.L.); 3 State Key Laboratory of Applied Optics, Changchun Institute of Optics, Fine Mechanics and Physics, Chinese Academy of Sciences, Changchun 130033, China

**Keywords:** GaN-based UV LED, wet chemical etching, prism-structured sidewall, crystal orientation, light extraction

## Abstract

We demonstrated that the tetramethylammonium hydroxide (TMAH) solution possesses different etching abilities to the chip sidewalls with different orientations because the orientation of chip sidewall determines the exposed crystallographic plane of gallium nitride (GaN) and these crystallographic planes are with different chemical stability to the TMAH solution. After TMAH etching treatment, trigonal prisms were observed on sidewalls where *m*-plane GaN was exposed. For the investigated two types of light-emitting diodes (LEDs) with orthogonal arrangements, the LEDs with their larger sidewalls orientated along the [11–20] direction exhibited an additional 10% improvement in light output power after TMAH etching treatment compared to the LEDs with larger sidewalls orientated along the [1–100] direction.

## 1. Introduction

Nitride-based LEDs have experienced significant progresses since the achievement of high-brightness GaN-based LEDs in the 1990s [1,2]. The ultraviolet LEDs (UVLEDs) are currently being extensively studied owing to their potential applications in air and water-purification, germicidal, and biomedical instrumentation systems [3]. However, the poor internal quantum efficiency and low light extraction efficiency constitute the bottlenecks in realizing high performance devices [4,5]. Improving the light extraction efficiency is considered to be critical for achieving high performance UVLEDs [6,7]. Due to the high refractive index of GaN with respect to the surrounding air, the critical angle for photons to escape from GaN layer is about 23.5° according to Snell’s law, and only little internal light can be extracted from the active layer for conventional nitride-based LEDs. Several methods have been proposed to improve the light extraction efficiency, such as chip geometry shaping [8,9], surface texturing [10,11], SiO_2_ current blocking layer [12], patterned sapphire substrate [13], photonic crystal [14], bottom reflector [15], and flip chip technology [16], patterned double-layer ITO [17]. Among the above mentioned methods, potassium hydroxide (KOH)-based wet etching of GaN to form textured surface is recognized as effective and low-cost and has attracted much research interest [18,19,20].

Generally, the KOH-based wet etching of GaN proceeds with the iterative processes of oxide formation in alkaline solution and subsequent dissolution of the oxide [21]. Without the aid of UV light irradiation or bias voltage, the wet etching only selectively occurs at semi-polar and non-polar planes of GaN, which would not prohibit the hydroxide ions from accessing the Ga atom [22,23,24]. It was also reported that the wet etching rate of GaN is dependent on the crystallographic planes of GaN [25,26,27]. Thus, the anisotropic wet etching of GaN generally leads to peculiar textures at different surfaces, such as trigonal prisms and hexagonal pyramids, by exposing specific crystallographic planes [21,28,29]. In the mass-production of LEDs, the LED chips are commonly cut into cuboid geometry by laser scribing without considering the sidewall orientation, which means that the exposed crystallographic planes are indecisive. Some researchers have reported that the surface morphologies are varied at different sidewalls for the striped GaN pattern and cuboid geometry LED chips after wet etching [20,25,27]. However, it is unknown whether the sidewall orientation has influence on the optical and electrical performance of LEDs with chemical etching treatment.

In this article, we intended to explore the influence of sidewall orientation on the performance of the GaN-based LEDs. A TMAH etching process instead of KOH etching was adopted since it can provide a more chemically stable etching process [30,31]. We first studied the surface morphologies of GaN epitaxial layers with different TMAH etching time. Selective etching of *m*-plane facet was demonstrated according to the surface morphologies of sidewalls with different orientations. Based on the selective etching phenomenon, we fabricated two types of cuboid LEDs, one with its two larger sidewalls parallel to the *a*-plane of sapphire and the other with its two larger sidewalls perpendicular to the *a*-plane of sapphire, to prove the importance of sidewall orientation for LEDs with wet etching treatment.

## 2. Materials and Methods

The GaN-based LEDs were grown on the *c*-plane of patterned sapphire substrates (PSS) with primary flat orientated in *a*-plane by using a metal-organic chemical vapor deposition (MOCVD) method. The epitaxial structures consisted of a 20 nm sputtered AlN nucleation layer, a 2.5-μm-thick undoped GaN buffer layer, a 2-μm-thick Si-doped n-GaN layer, a 12 pair In_0.02_Ga_0.98_N (2.5 nm)/GaN (3 nm) superlattice layer, a 10 pair of In_0.07_Ga_0.93_N (3 nm)/GaN (12 nm) multiple quantum well (MQW) active layer, a 20 nm-thick p-Al_0.2_Ga_0.8_N electron blocking layer, and a 200-nm-thick Mg-doped p-GaN layer. The LED wafer was subsequently annealed at 750 °C at N_2_ atmosphere to activate Mg acceptor in the p-GaN.

The GaN epitaxial layer was etched by inductively-coupled plasma (ICP) based on BCl_3_/Cl_2_ mixture gas to form mesa structure and expose the n-GaN layer. Samples were then dipped into the 15wt% TMAH solution at 85 °C to conduct the TMAH-based crystallographic etching procedure after ICP etching. A 230-nm-thick indium tin oxide (ITO) was deposited on the p-GaN as a p-type ohmic contact using electronic beam evaporator, followed by thermal annealing at 550 °C under N_2_ ambient conditions. Next, Cr/Pt/Au (10 nm/25 nm/1.2 μm) metal was deposited on the ITO and n-GaN layers to form the p- and n- electrodes; Finally, the UVLED wafers were thinned down to be about 130 μm and diced into chips with dimension of 712 μm × 254 μm. The peak wavelength of UVLEDs is 395 nm. Light output power–current-voltage (*L-I-V*) characteristics of UVLEDs were measured using a calibrated integrating sphere and a semiconductor parameter analyzer (Keysight B2901A, Santa Rosa, CA, USA). 

**Figure 1:**
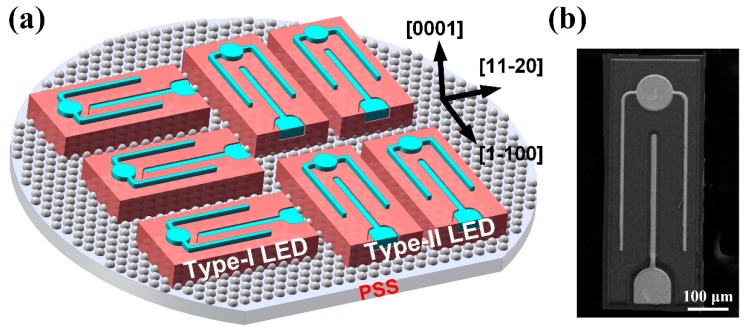
(**a**) Schematic diagram of Type-I LED and Type-II LED with different sidewall orientations. (**b**) SEM image of the fabricated LED chip.

In this work, we kept the size of device area at 712 μm × 254 μm unchanged, while the chip sidewall orientation was varied as shown in Figure 1. We named the two types of LEDs as Type-I LED and Type-II LED, respectively. The larger sidewalls of Type-I LED were set to be parallel to the *a*-plane of sapphire substrate while the larger sidewalls of Type-II LED were set to be perpendicular to the *a*-plane of sapphire substrate.

## 3. Results and Discussion

**Figure 2:**
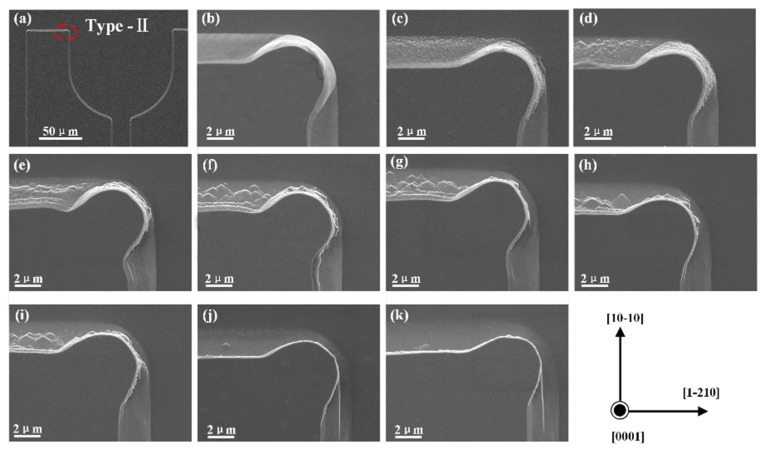
(**a**) SEM images of Type-II LED chips, the marked corner was chosen to show the morphology change after TMAH etching. (**b**–**k**) SEM images of the marked corner with different etching time: (**b**) 0 min; (**c**) 1 min; (**d**) 2.5 min; (**e**) 5 min; (**f**) 7.5 min; (**g**) 10 min; (**h**) 15 min; (**i**) 20 min; (**j**) 30 min; and (**k**) 40 min.

The morphologies of epitaxial layers with different TMAH etching time were investigated with a TESCAN MIRA 3 LMH field emission scanning electron microscope (SEM, TESCAN, Brno, Czech Republic). As shown in Figure 2a, the marked corner was chosen to show the morphology change after TMAH etching for the reason that the corner structure presented more morphology information with three perpendicular sidewalls. Within the investigated TMAH etching time, trigonal prism structures were observed on the sidewall along the [1–210] direction (with the exposed m-plane), while no such structure appeared on the top surface of p-GaN (with the exposed + c-plane) and the sidewall along the [1–100] direction (with the exposed *a*-plane). This phenomenon indicated that the TMAH solution possesses different etching ability to the exposed crystallographic plane. The TMAH etching starts with the attachment of hydroxide ions to the positively-charged Ga dangling bonds, while adjacent negatively-charged N dangling bonds would prohibit the hydroxide ions from accessing Ga atom. Thus, the density of N dangling bonds on each crystallographic plane determines the etching ability of TMAH solution. As previously reported, the density of N dangling bonds can be ranked as follows: +*c*-plane > *a*-plane > *m*-plane [26]. The calculated density of N dangling bonds suggested that TMAH has the largest etching ability to *m*-plane among the three listed crystallographic planes, which may explain why the TMAH etching did not occur on the top surface of p-GaN and *a*-plane sidewall. The size and density of trigonal prisms on the *m*-plane sidewall varied with different TMAH etching time. With a short TMAH etching time of 1 min, miniature prisms formed throughout the *m*-plane sidewall of n-GaN as shown in Figure 2c. Increasing the TMAH etching time led to the coalescence of miniature prisms to generate larger trigonal prisms (Figure 2d–f). However, the density of trigonal prisms decreased with increasing TMAH etching time when the TMAH etching time was over 7.5 min (Figure 2g,j). A TMAH etching treatment of 40 min finally led to the disappearance of trigonal prism (Figure 2k). It was proposed that the density of trigonal prisms is first–order inversely proportional to the etching time for a given etchant concentration and temperature owing to that the wet chemical etching was demonstrated a first-order reaction [28,32], which means that the etching rate is also inversely proportional to the density of prisms. According to the Arrhenius equation:

(1) $$r=r_{0}e^{-E_{a}/kT}$$

where *r* is the etching rate, *r_0_* is a constant, *E_a_* is the activation energy, *k* is Boltzmann’s constant, and *T* is the temperature. Substituting the etching rate in Equation (1) with the density of trigonal prisms (ϱ):

(2) $$\frac{1}{\varrho }=\frac{1}{\varrho_{0}}e^{{-E}_{a}/kT}$$

**Figure 3:**
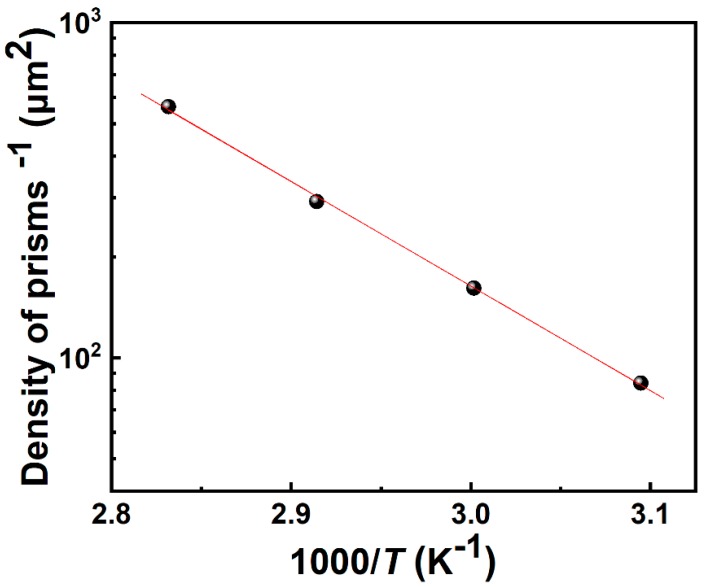
The inverse density of prisms plotted as a function of the reciprocal of temperature following the Arrhenius plot fit.

To obtain the activation energy of the TMAH etching reaction, the inverse of density was plotted as a function of the inverse of the temperature as shown in Figure 3. The activation energy was finally determined to be 25.9 kcal mol^−1^, which is slightly larger than that of KOH etching (21 kcal mol^−1^) [32].

**Figure 4:**
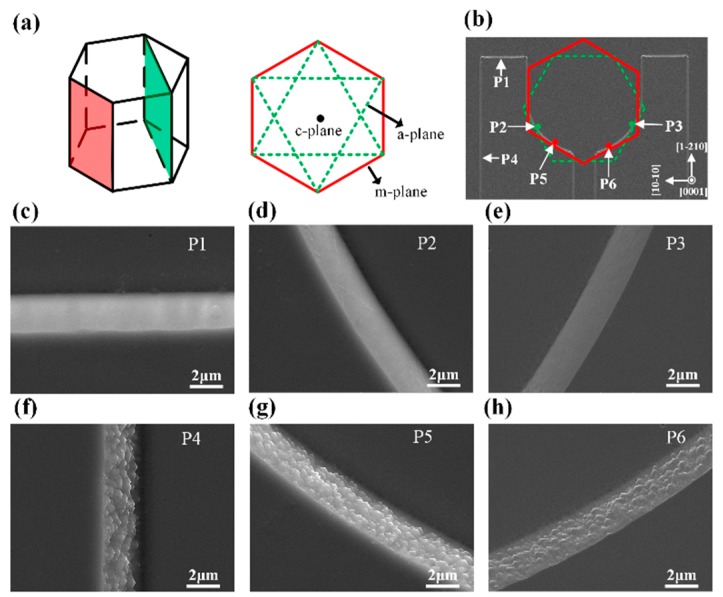
(**a**) Schematic of the wurtzite structure GaN unit cell and its projection in the *c*-plane. (**b**) SEM images of Type-I LED chips, the projection was incorporated to show the exposed *m*-plane and *a*-plane. (**c**–**h**) The SEM images at marked positions: (**c**) P1; (**d**) P2; (**e**) P3; (**f**) P4; (**g**) P5; and (**h**) P6.

The selective etching phenomenon was further demonstrated from the SEM images of Type-I LED with etching time of 2.5 min as shown in Figure 4. We chose sidewalls with different orientations to illustrate the selective etching of *m*-plane sidewall. Figure 4a is the GaN unit cell projection of hexagonal wurtzite structure, only the *m*-plane and *a*-plane were outlined because we mainly focused on the different etching phenomena between the *m*-plane and the *a*-plane. By incorporating the projection into the SEM image, we can mark the exposed *m*-plane and *a*-plane. As shown in Figure 4b, the sidewalls at the P1, P2, and P3 regions with different orientations were ascribed to the *a*-plane sidewall, while the sidewalls at the P4, P5, and P6 regions with different orientations were ascribed to the *m*-plane sidewall. For the Type-I LED with a 2.5 min TMAH etching process, no trigonal prism was observed at the P1, P2, and P3 regions while trigonal prisms formed throughout the P4, P5, and P6 regions. The result further validated that the TMAH selectively etched the *m*-plane sidewall to generate trigonal prisms.

**Figure 5:**
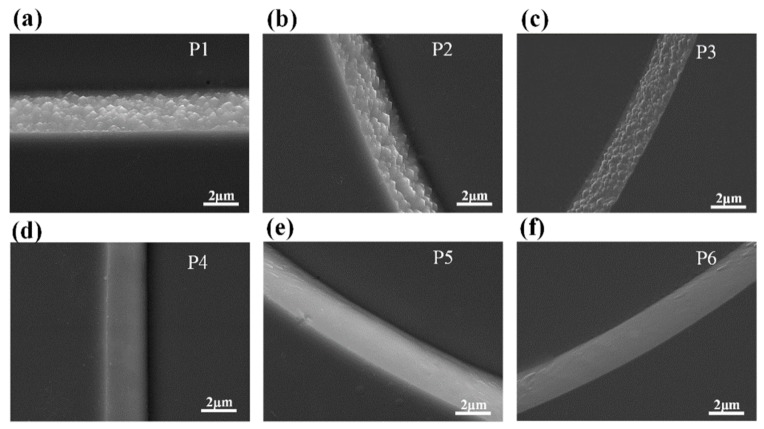
(**a**–**f**) SEM images of selected areas of Type-II LED chips, (**a**) P1; (**b**) P2; (**c**) P3; (**d**) P4; (**e**) P5; and (**f**) P6. The six regions were chosen at the same position as they were in the Type-I LED chips.

Figure 5 shows the SEM images of Type-II LED with 2.5 min TMAH etching. The presented six regions were chosen at the same position as they were in the Type-I LED. Due to the different orientations of the two types of LEDs, the exposed crystallographic plane in the same area is changed. Contrary to that in the Type-I LED, the sidewalls at the P1, P2, and P3 regions were ascribed to the *m*-plane sidewall while the sidewalls at the P4, P5, and P6 regions were ascribed to the *a*-plane sidewall. Due to the different TMAH etching phenomena between *m*-plane sidewall and *a*-plane sidewall, reversed rough sidewalls were generated in the Type-II LED relative to the Type-I LED.

**Figure 6:**
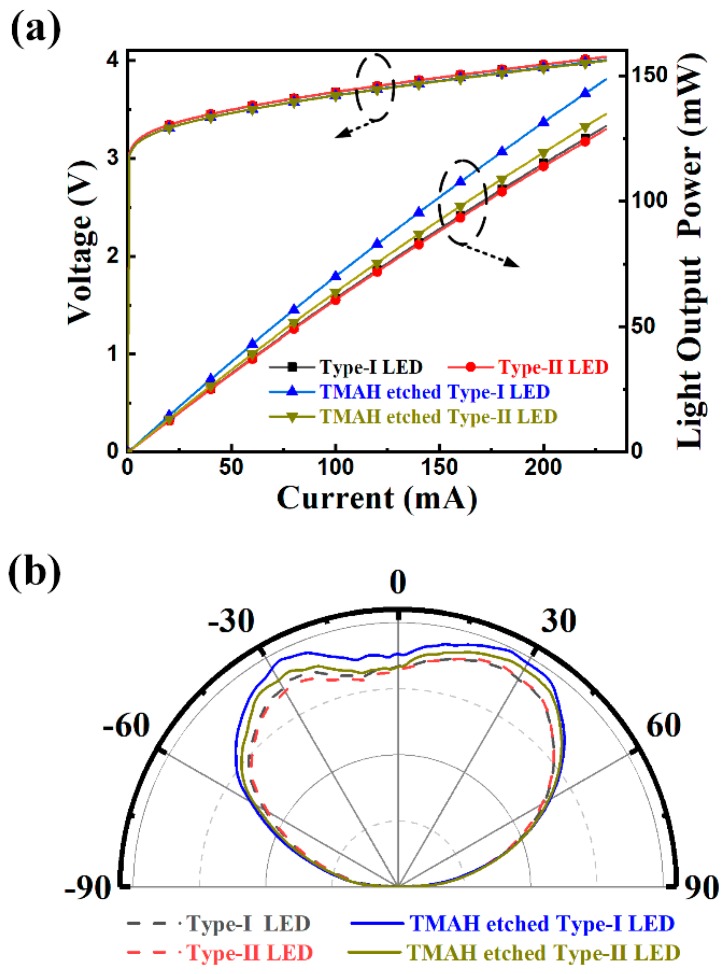
(**a**) *L-I-V* characteristics of the cuboid geometry LEDs with and without TMAH etching treatment. (**b**) Far-field emission patterns of the cuboid geometry LEDs with and without TMAH etching treatment.

Figure 6a showed the *L-I-V* characteristics of the two types of LEDs with and without TMAH treatment. Without TMAH etching treatment, the investigated two types of LEDs showed almost the same electrical characteristic. After TMAH etching treatment, both of the two types of LEDs showed a 0.3 V decrease in forward voltage. The decreased forward voltage was due to better ohmic contacts between ITO transparent conductive electrode and p-GaN in the TMAH etched LED chips. The TMAH etching process can eliminate the oxide on the p-GaN surface and improve the carrier density near the p-GaN surface as previously reported [30], which are beneficial for the improvement of ohmic contact. With injection current of 60 mA, the light output powers for the TMAH etched type-I LED and TMAH etched Type-II LED were 42.99 mW and 39.13 mW, which were improved by 15% and 5% as compared to the un-etched LED. Although it has been reported that TMAH etching effectively improve the LED performance [30,33], it is unaware that the orientations of LED chip sidewalls play a significant role in the etching process. It is noteworthy that the TMAH etching process created reversed rough sidewalls for the two types of LED chips in our experiments. For the cuboid geometry chips as shown in Figure 1, Type-I LED chips possess larger rough sidewalls than that of Type-II LED chips after the TMAH etching process. The textured surfaces created by wet chemical etching can efficiently improve the opportunities that photons incident into the escape cone, leading to an increase in light extraction efficiency. Figure 6b shows the far-field radiation profiles of the investigated LEDs measured at the injection current of 50 mA. Compared to the unetched LEDs, the TMAH etched LEDs exhibited an enhanced emission intensity in the ±[15°, 60°] direction, suggesting that more photons escaped from sidewall. The TMAH etched Type-I LED had the strongest emission intensity among the investigated LEDs, demonstrating that larger textured sidewall is beneficial for light extraction.

## 4. Conclusions

To summarize, we studied the TMAH etching process in UVLEDs. The TMAH selectively etched the exposed the *m*-plane sidewall of GaN to form trigonal prisms in the sidewalls. By varying the orientation of chip sidewall, the exposed crystallographic plane can be changed and, thus, the textured sidewall resulting from TMAH etching can be adjusted. For the cuboid geometry LED chips investigated in our experiments, the LED chips with larger *m*-plane sidewalls gave the best light output power after TMAH etching. Our work revealed the significance of sidewall orientation in wet chemical etched LEDs, which is meaningful in the mass-production of high-performance LEDs.
